# Optimizing diagnosis and management of patients with ENPP1 deficiency: an expert opinion

**DOI:** 10.1007/s40618-026-02843-1

**Published:** 2026-03-02

**Authors:** Irene Bruno, Luisa De Sanctis, Nadia Edvige Foligno, Federica Giambò, Stefano Mora, Mattia Parolin, Giuseppe Vezzoli

**Affiliations:** 1 Metabolic and Rare Diseases Unit, IRCCS Burlo Garofolo, Trieste, Italy; 2 Pediatric Endocrinology, Regina Margherita Children’s Hospital, Turin, Italy; 3 Department of Public Health and Pediatric Sciences, University of Turin, Turin, Italy; 4 Nephrology and Dialysis Unit, IRCCS San Raffaele Hospital, Vita Salute San Raffaele University, via Olgettina 60, Milan, 20132 Italy; 5 Laboratory of Pediatric Endocrinology, Department of Pediatrics, IRCCS San Raffaele Hospital, Milan, Italy; 6 Pediatric Nephrology, Dialysis and Transplantation Unit, University Hospital, Padova, Italy

**Keywords:** Autosomal recessive hypophosphatemic rickets type 2, Ectopic calcification disorders, ENPP1 deficiency, Generalized arterial calcification of infancy type 1, Patient journey

## Abstract

**Purpose:**

Ectonucleotide pyrophosphate/phosphodiesterase family member 1 (ENPP1) deficiency is a rare genetic disorder caused by loss-of-function *ENPP1* gene mutations. Characterized by abnormally low circulating inorganic pyrophosphate concentrations, bone hypomineralization, soft tissue calcification, and arterial stenosis, ENPP1 deficiency is associated with a phenotypic spectrum that includes generalized arterial calcification of infancy type 1 (GACI1) and autosomal recessive hypophosphatemic rickets type 2 (ARHR2). Despite phenotypic differences, patients with GACI1 and ARHR2 have a marked, lifelong physical and emotional burden, with a negative impact on quality of life.

**Methods:**

A scientific board, comprising seven specialist physicians (endocrinologists, nephrologists, and pediatricians) practicing in Italy, held two virtual meetings to exchange knowledge regarding real-world clinical experience of GACI1 and ARHR2 using case examples, and to discuss strategies on how to increase disease awareness and optimize the diagnosis and management of these patients.

**Results:**

Five real-world clinical cases are described. The specialist physicians also provide guidance for optimizing the management pathway for patients with ENPP1 deficiency. Early identification of the clinical signs of disease, in combination with comprehensive diagnostic and follow-up testing, is essential for effective patient management.

**Conclusion:**

Early and accurate identification of ENPP1 deficiency by healthcare providers and comprehensive diagnostic testing is essential for the effective management of patients with GACI1 and ARHR2. Consistent follow-up is key to preventing complications and adverse outcomes. Although treatment options are limited, novel therapies are currently under clinical development.

## Introduction

**Fig. 1:**
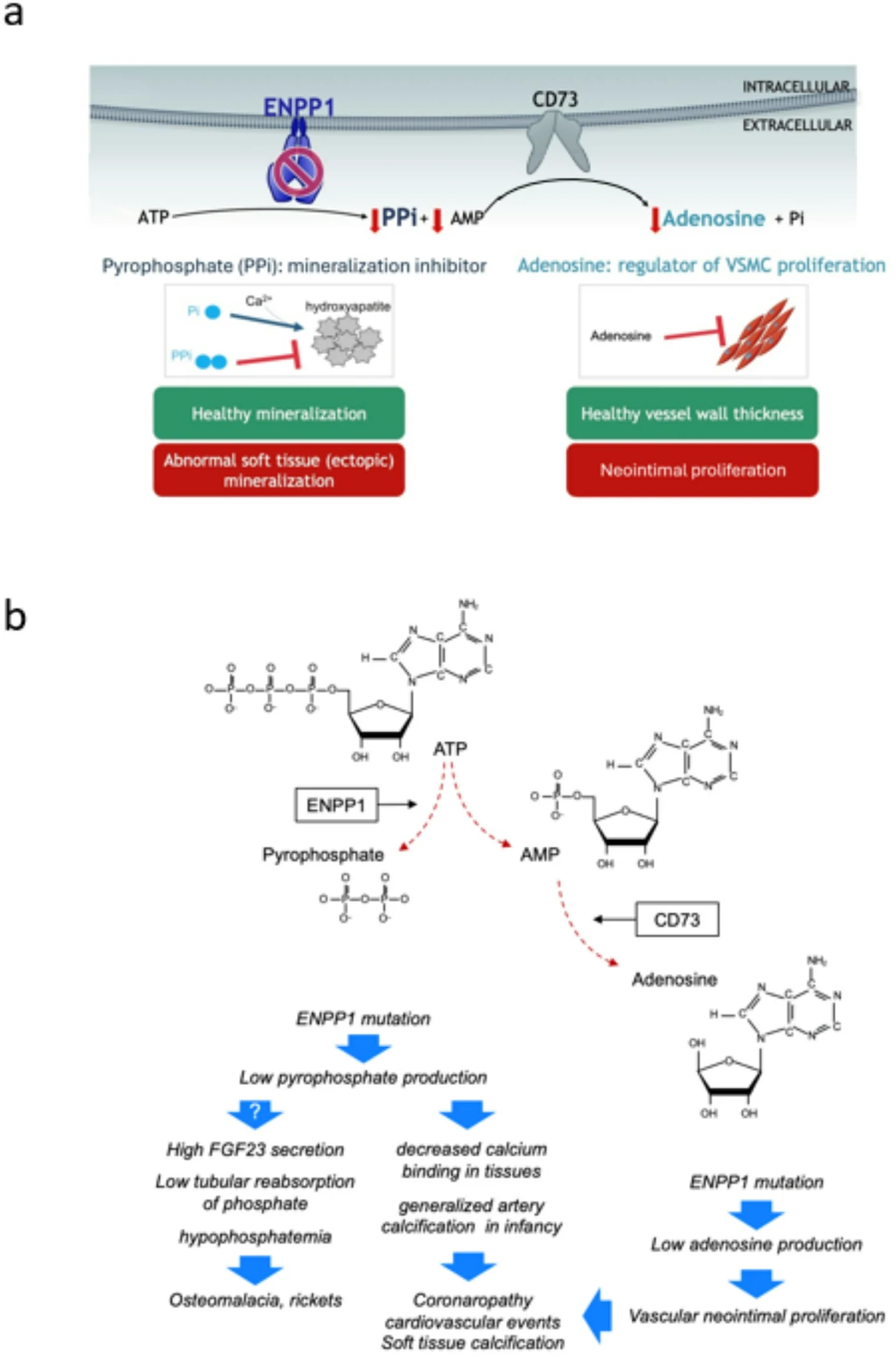
ENPP1, encoded by ENPP1 gene, is a transmembrane enzyme that cleaves ATP into AMP and diphosphate/pyrophosphate (panel a). Mutations in ENPP1 gene causing ENPP1 loss of function (indicated by a crossed out red circle) have been associated with GACI and hypophosphatemic rickets autosomal recessive 2 (ARHR2) (panel b). ARHR2 is mediated by an increase in FGF23 secretion which is sustained by an unknown mechanism (panel b). After its production mediated by ENPP1, AMP may be cleaved into adenosine and phosphate by CD73 [5], an endogenous 5′-nucleotidase. Therefore, ENPP1 inactivation leads to downregulation of adenosine production and vascular smooth muscle cells proliferation that may contribute to the arterial damage in GACI (panel a and b). Adapted from Inorganic pyrophosphate deficiency syndromes and potential treatments for pathologic tissue calcification (Ralph D, et al. [2022]. Am J Pathol 192:762–770). Reproduced with permission by Elsevier (https://pmc.ncbi.nlm.nih.gov/articles/PMC9088198/). Abbreviations: AMP: adenosine monophosphate; ARHR2: autosomal recessive hypophosphatemic rickets type 2; ATP: adenosine triphosphate; ENPP1: Ectonucleotide pyrophosphatase/phosphodiesterase 1; FGF23: fibroblast growth factor 23; GACI1: generalized arterial calcification of infancy type 1; Pi: inorganic phosphate; PPi: inorganic pyrophosphate; VSMC: vascular smooth muscle cell

Ectonucleotide pyrophosphatase/phosphodiesterase family member 1 (ENPP1) is a key enzyme involved in the generation of inorganic pyrophosphate (PPi) [1], which plays an essential regulatory role in soft tissue calcification and bone mineralization (Fig. 1) [2]. The ENPP1 catalytic domain cleaves the phosphodiester bonds of nucleotides, preferentially hydrolyzing extracellular adenosine triphosphate (ATP) into PPi and adenosine monophosphate, the latter of which is further metabolized to adenosine [3]. Loss-of-function *ENPP1* mutations result in ENPP1 deficiency, which is characterized by abnormally low circulating PPi concentrations, bone hypomineralization, soft tissue calcification, and arterial stenoses [3]. Persistent stenosis is thought to be attributed to reduced amount of adenosine, which normally acts as an inhibitor of neointimal proliferation [4].

ENPP1 deficiency is a rare disorder, with prevalence estimates varying between 1 in 64,000–200,000 pregnancies [6, 7]. Bi-allelic ENPP1 deficiency is associated with a phenotypic continuum of abnormal mineralization that includes generalized arterial calcification of infancy type 1 (GACI1) and autosomal recessive hypophosphatemic rickets type 2 (ARHR2) [8–12]. GACI1 is characterized by *in utero*- or infantile-onset, widespread vascular calcifications that cause multiple cardiovascular complications and high infant mortality [13, 14]. The majority of patients with GACI1 who survive beyond infancy will develop ARHR2 in childhood [7]. Throughout their lives, individuals with GACI1 may also experience periarticular calcification because PPi inhibits hydroxyapatite crystal deposition, thereby resulting in extra-articular calcifications [1]; hearing loss and cervical spine fusion can also occur [7]. ARHR2 can be found in GACI1 survivors, but it may also be the first manifestation of ENPP1 deficiency. It is characterized by renal phosphate wasting and hypophosphatemic rickets leading to osteomalacia, with defects in bone mineralization and bone deformities that manifest during early childhood [11, 15, 16]. The initial trigger of these alterations is the overproduction of fibroblast growth factor 23 (FGF23) associated with ENPP1 deficiency [11, 15, 17]. FGF23 functions as the central regulator of phosphate metabolism by increasing the renal excretion of phosphate, thereby escalating renal phosphate wasting [18].

Both GACI1 and ARHR2 are caused by a spectrum of loss-of-function *ENPP1* mutations [8, 15, 19, 20]. Despite the phenotypic heterogeneity between these disorders, all patients with ENPP1 deficiency experience lifelong complications that cause significant physical and emotional burdens and poor quality of life for patients and their caregivers [21]. In our clinical experience, if children survive beyond the first 6 months of life, their prognosis depends on comorbidities that develop during this period (such as cardiac or neurological complications), as well as on the subsequent course of the disease and any complications secondary to conventional therapy.

The purpose of this article is to increase awareness of GACI1 and ARHR2 by providing real-world clinical experience from specialist physicians (endocrinologists, nephrologists, and pediatricians) and describing their opinions on how to optimize the diagnosis and management of patients living with these conditions.

## Methods

The scientific board, set up on the initiative of Professor Vezzoli to share knowledge and clinical experiences from clinical centers managing patients with ENPP1 deficiency in Italy, was composed of seven specialist physicians with experience in the field of GACI1/ARHR2 patient management (the authors of this review), who met in two virtual meetings on November 28, 2023 and March 25, 2024 to exchange knowledge regarding their clinical experience and to discuss strategies for increasing disease awareness among other physicians. Prior to these meetings, a search of PubMed was conducted on November 8, 2023 using the search terms ‘*ENPP1 deficiency*, ‘*generalized arterial calcification of infancy (GACI)*’, ‘*autosomal recessive hypophosphatemic rickets type 2 (ARHR2)*’, ‘*ectopic calcification*’, ‘*ABCC6 deficiencies*’, ‘*arterial calcification*’, and ‘*biomarkers*’ to identify relevant publications. Other articles were included based on the authors’ publication knowledge in the relevant areas.

### Clinical signs of disease

#### GACI1

GACI1 may present as a wide spectrum of overlapping phenotypes, characterized by soft tissue calcification and cardiovascular involvement (Table 1) [7, 22, 23]. In neonates, GACI1 usually presents with a severe phenotype that includes pericardial effusion, heart failure, severe hypertension, fetal hydrops, and a sepsis-like condition. Patients with GACI1 have characteristic vascular calcifications that may be identified during prenatal screening or at any time after birth and that may subsequently disappear or decrease, either spontaneously within 2–3 years of life [24] or with treatment; only arterial stenoses do not regress spontaneously or with treatment in the majority of cases [25]. In contrast, patients who develop symptoms later in life have a milder phenotype, including rickets, delayed loss of deciduous teeth, and musculoskeletal disorders, but with a minimal tendency to develop calcifications [24].

**Table 1:** The prenatal and postnatal clinical signs of GACI1 and ARHR2

	GACI1	ARHR2
Prenatal signs	Family health history of perinatal death	Family health history of perinatal death
	Family history of death within the first year of life	Family history of death within the first year of life
	Consanguinity	Consanguinity
	Prenatal calcification of large and small arteries	Prenatal calcification of large and small arteries
	Heart failure	
	Fetal hydrops	
	Polyhydramnios	
	Pericardial effusion	
Postnatal signs	Heart failure	Rickets
	Sepsis-like condition	Lower limb deformation
	Hypertension	Hypophosphatemia
	Cardiovascular instability	Disharmonious short stature
	Cardiomegaly	Delayed walking
	Respiratory distress	Abnormal gait
	Pericardial effusion	Muscle pain or fatigue when walking
	Ectopic calcifications	Enthesitis
	Hypophosphatemia	Calcification of tendons, ligaments and joints
	Rickets during the first years of life	Calcification of stapes and hypoacusis
	Kidney failure	Calcification or stenosis of large and medium-sized arteries and valves
		Bone malformations in early childhood
		Late loss of deciduous teeth
		Kidney failure
		Nephrocalcinosis
		Kidney stones

Abbreviations: ARHR2: autosomal recessive hypophosphatemic rickets type 2; GACI1: generalized arterial calcification of infancy type 1

The prenatal/perinatal period is important for the identification of neonatal disease. The prenatal signs of GACI1 include a family history of perinatal death or death within the first year of life, consanguinity, heart failure, fetal hydrops, echogenicity of major arteries, pericardial effusion, and polyhydramnios on prenatal ultrasound [26–32]. The postnatal signs of GACI1 may include a sepsis-like condition, hypertension, cardiovascular instability, calcification of blood vessels and organs, intimal hyperplasia and/or arterial stenosis, peripheral edema, joint calcification, feeding difficulties, respiratory distress, and hypophosphatemia [10, 26, 28, 30, 33–35]. Patients with GACI1 may also present with acute kidney injury [36] or develop mild-to-moderate hearing loss [37]. In neonates with these signs, ultrasound, X-ray, and electrocardiogram further aids in confirming the diagnosis.

To optimize patient outcomes, it is essential to diagnose GACI1 during the first 6 months of life, not only for cases requiring immediate treatment, but also for those who initially present with calcifications without hypertension or heart failure that later resolve. In the latter group of patients, there is a risk of a delayed diagnosis of rickets, extra-articular calcifications, or short stature, and these patients may be lost to follow-up.

#### ARHR2

Similar to GACI1, the prenatal signs of ARHR2 include a family history of perinatal death or death within the first year of life, consanguinity, and calcification of large and small arteries (Table 1). ARHR2 typically presents in early childhood with rickets, lower limb deformation, short stature, delayed walking, abnormal gait, bone pain, fatigue, cervical spine fusion, premature fusion of skull bones (craniosynostosis), calcific enthesopathy, enthesitis, hearing loss, and delayed loss of deciduous teeth. These disorders are associated with low phosphate excretion threshold and hypophosphatemia secondary to high FGF23 production and increased alkaline phosphatase (ALP), especially its bone specific fraction [15, 17, 28, 38, 39]. Patients with ARHR2 may also have calcification and stenosis of large and medium arteries and valves [10]. These calcifications may develop in infancy but remain undetected in patients who are diagnosed with ARHR2 later in life [24].

### Real-world clinical experience

During the two meetings, the authors discussed their experience with five clinical cases of ENPP1 deficiency that they had treated in routine clinical practice (Table 2). The following section provides an overview of these patients and highlights the key clinical characteristics of this rare enzyme deficiency.

**Table 2:** Summary of real-world clinical cases of ENPP1 deficiency

Case		Clinical manifestations	Primary phenotype
1		Hypophosphatemic rickets (childhood onset) Periarticular calcifications on hands and feet Nephrocalcinosis Aortic steno-insufficiency Hypercalciuria Secondary or tertiary hyperparathyroidism	ARHR2
2		Fetal hydrops (pre-natal) Treatment-refractory systemic hypertension (at birth) Arterial and periarticular calcifications (at birth) Cardiac arrest (neonatal period) Spastic quadriplegia (in 2nd year of life) Hypophosphatemia (at 2 years)	GACI1
3		Valvular and abdominal aortic calcifications (pre-natal) Arterial hypertension (in 1st month of life) Left ventricular hypertrophy and aortic annulus calcifications (at 3 months) Skeletal dysplasia, calcifications of posterior cervical joints, auricles and annular ligaments of stapes, hypoacusis, keratosis (in childhood)	GACI1 + ARHR2
4		Bilateral hip dysplasia (infant-onset) Delayed walking Parietal bumps, rachitic rosary, rachitic changes of the wrist, femorotibial joint varus (at 3 years) Reduced growth at diagnosis Systolic 2/6 murmur, aortic valve calcification Delayed loss of deciduous teeth Transmissive hypoacusis, psoriasis Midfoot pronation with femoral anteversion	ARHR2
5		Cardiac flow and hepatic hyperechogenicity, cardiac effusion (pre-natal) Arterial calcifications (calcified coronary artery and aorta with a narrowing), pericardial effusion, severe arterial hypertension (at birth) Coxofemoral joint calcification (at 2.5 months)	GACI1

Abbreviations: ARHR2: autosomal recessive hypophosphatemic rickets type 2; ENPP1: ectonucleotide pyrophosphatase/phosphodiesterase family member 1; GACI1: generalized arterial calcification of infancy type 1

#### Case 1

##### Case 1

is a female patient who was diagnosed with hypophosphatemic rickets at the age of 2 years in 1985 due to the appearance of genu valgum. She was treated with calcifediol, later replaced with calcitriol, and phosphates. Her subsequent clinical history included multiple limb lengthening surgical treatments between the ages of 6 and 18 years; appearance of periarticular calcifications on the hands and feet to calcific enthesitis (11 years); mild nephrocalcinosis initially identified on kidney ultrasound (12 years) that remained stable on follow-up ultrasound (19 years); mild-to-moderate aortic steno-insufficiency; and normal serum calcium and hyperparathyroidism, without parathyroid nodules at neck sonography detected at the age of 22 years. It was concluded that the patient’s conventional treatment had led to hyperparathyroidism, as well as calcification of her tendons and renal pyramids.

An *ENPP1* mutation (homozygous for IVS22 + 1G > A) was confirmed by molecular analysis (25 years) and the patient had continued therapy with calcitriol only. At the age of 26 years, she developed mild hypercalcemia (calcium 2.62 mmol/L; normal range 2.2–2.6) associated with high plasma parathyroid hormone (PTH; 70 pg/mL; normal range 15–65), which persisted despite the withdrawal of calcitriol; hypercalcemia was attributed to tertiary hyperparathyroidism due to chronic oral phosphate supplementation. In addition, serum phosphate (0.45 mmol/L; normal range in adults 0.8–1.5), vitamin D (19.5 ng/mL; normal range 20–68), 1,25-dihydroxyvitamin D (20.1 pg/mL; normal range 20–80), and tubular reabsorption of phosphate (75%; normal range 80–90%) were low; ALP was normal (83 U/L; normal range 33–98). Neck ultrasound detected a hypoechoic parathyroid nodule; however, methoxy-isobutyl-isonitrile (MIBI) scintigraphy was negative for parathyroid nodules and her spine and total body bone mineral density was normal. Her serum FGF23 was measured for the first time at the age of 22 years with a Kaynos kit (39 pg/mL; normal range 10–50 pg/mL) when she also had hypophosphatemia (phosphate 0.45 mmol/L) and was treated with calcitriol. The second time was in 2020 when her serum FGF23 was measured with a Diasorin kit (75 pg/mL; normal range 20–90); she also had hypophosphatemia on this occasion (0.53 mmol/L) and therapy with paricalcitol was started.

She was treated with cinacalcet after the suspension of calcitriol to maintain normal serum calcium levels. Her clinical picture remained stable for approximately 1 year, at which time, cinacalcet was withdrawn before the patient became pregnant. Hypercalcemia did not recur after withdrawal of cinacalcet and paricalcitol was later started in an attempt to inhibit parathyroid activity more effectively than calcitriol [40].

The clinical picture of Case 1 more closely resembled ARHR2 than GACI. It suggests that secondary or tertiary hyperparathyroidism may develop in patients with ENPP1 deficiency, as previously reported in a case series of a family with monoallelic and biallelic *ENPP1* mutations [22]. This case highlights how cinacalcet treatment could effectively reduce the significance of the hypertrophic damage of the parathyroid gland, as reported in patients with chronic kidney disease [41]. Of note, this patient achieved partial recovery of parathyroid function, particularly during pregnancy, and her child was not affected by ENPP1 deficiency.

#### Cases 2 and 3

##### Cases 2

and 3 are female siblings with GACI1 of differing severity; the details of these cases have been previously described [42].

##### Case 2

had severe treatment-resistant arterial hypertension immediately after her birth at 31 weeks of gestation via caesarian section (due to acute fetal distress and fetal hydrops) in 1993. X-ray imaging showed extensive arterial and periarticular calcifications and she was clinically diagnosed with GACI1. After 3 months, prostaglandin treatment was successfully discontinued and antihypertensive medications were significantly reduced. The arterial calcifications had spontaneously resolved by 6 months of age. During her second year of life, she developed spastic quadriplegia, most likely as a long-term sequela of a cardiac arrest requiring resuscitation and mechanical ventilation in the neonatal period. At 2 years of age, she developed severe hypophosphatemia (phosphate 0.93 mmol/L; normal range 1.25–2.10) without any radiologic signs of rickets. Despite marked hypophosphatemia as low as 0.80 mmol/L, she had normal ALP, PTH, vitamin D metabolites, and urinary β2-microglobulin. Genetic analysis at 10 years of age found missense *ENPP1* gene mutations c.913 C > A and c.1164 + 2T > A. At 11 years of age, her hypertension was well controlled with enalapril and echocardiography showed diffuse left ventricular hypertrophy and mild right ventricular hypertrophy, with minimal signs of mitral annulus calcification. At the latest follow-up, Case 2 was 31 years old. Her clinical status is characterized by persistent spastic quadriplegia, ongoing extra-articular calcifications (specifically multifocal ligamentous calcific appositions at C4–C5, meniscal calcifications, and periacetabular and peripatellar calcifications), and mild hypertension, managed solely with an angiotensin-converting enzyme (ACE) inhibitor.

##### Case 3

was born in 2001, 8 years after her sister. During pregnancy, ultrasound detected mitral valve echogenic foci in the left ventricle (at 14 weeks of gestation), then increased echogenicity and thickening of the abdominal aortic wall proximal to the iliac bifurcation (at 26 weeks of gestation), and hyperechogenicity of the aortic and pulmonary valve annulus with normal intracardiac flow (at 36 weeks of gestation). The infant was born at 37 weeks of gestation by caesarian section, with low (< 10th percentile) birth weight (2200 g), head circumference and length (31 cm and 47.5 cm, respectively), and Apgar scores of 8 and 9 at 1 and 5 min, respectively. Echocardiography at birth confirmed the prenatal findings of left ventricular hypertrophy, and aortic annulus calcification, and a chest/abdominal X-ray showed abdominal aortic calcification; she was diagnosed with GACI1. In the first month of life she developed arterial hypertension that was well controlled with captopril treatment. At 8 months of age, left ventricular hypertrophy had progressed, whereas the calcifications had disappeared, and there were no periarticular or arterial calcifications. At 3 years of age, mutational analysis of the *ENPP1* gene showed she had the same genotype as her sister. She did not develop hypophosphatemia. A bone mineral density scan was performed and was normal. At last follow-up (21 years of age), the patient had skeletal dysplasia (short stature; 142 cm tall), but she had no neurologic symptoms. However, she had calcifications of the posterior cervical joints, auricles, and annular ligaments of stapes, with mild conductive hearing loss in both ears. In November 2023, Case 3 entered a clinical trial (NCT04686175) for an investigational drug, INZ-701, as ENPP1 enzyme replacement therapy.

##### Cases 2

and 3 highlight that neonatologists, midwives, gynecologists, and prenatal sonographers need to be aware of GACI1, and that early diagnosis may improve neonatal survival. These cases also demonstrate that siblings with the same genotype can show variability and different severity in the clinical phenotype. Detection of prenatal calcifications does not necessarily mean that there will be neonatal disease onset. This phenotypic heterogeneity, as observed in many rare genetic diseases, depends on the genetic background, as well as modifier genes and epigenetic factors [43].

#### Case 4

##### Case 4

involves a female patient who presented with lower limb and muscle pain at the age of 3 years and 7 months. Her medical history included delayed walking, bilateral hip dysplasia (treated with a retractor), and vitamin D treatment for 3 months after birth. At the time of her first referral, the physical examination revealed parietal bumps, a rachitic rosary, rachitic changes in the wrist, and femorotibial joint varus. Her height was 94.3 cm (25th percentile) and weight was 14.5 kg. A lower limb X-ray revealed radiologic findings consistent with rickets, including curved femurs, with an enlarged and irregular distal metaphysis. The evaluation of calcium-phosphorus metabolism showed elevated ALP activity (527 UI/L; normal range 150–380), hypophosphatemia (phosphate 0.83 mmol/L; normal range in children 1.3–1.8), and a low 25-hydroxyvitamin D concentration (16.7 ng/mL; normal > 30 ng/mL), with normal calcium (2.35 mmol/L; normal range 2.1–2.5), PTH (52.7 pg/mL; normal range 9–65), and 1,25-dihydroxyvitamin D levels (24 pg/ml; normal range 20–80 pg/ml); tubular reabsorption of phosphate (82%) was within the normal range. After the correction of 25-hydroxyvitamin D deficiency, the patient started conventional treatment with phosphate salts and calcitriol. A systolic 2/6 murmur was detected, and echocardiography revealed a thickening of the aortic valve cusp, with possible calcification and pseudofusion of the cusps, hypomobility of the right cusp, minimal anterograde acceleration, and mild valve insufficiency. Given these findings, hereditary hypophosphatemic rickets was suspected and genetic analysis detected *ENPP1* mutations (c.715 + 2T > g,mat trans variation and c.I437+3_I437 + 6del4,pat deletion). At the time of the diagnosis of hypophosphatemia, testing for FGF23 was not available.

During clinical follow-up, echocardiography showed stable aortic valve calcification and cardiac function, with no need for treatment. Over time, the patient reported fatigue and pain in the thighs, but experienced regular growth with improved height velocity, progressing from the 25th to the 50th percentile. She also developed tibial varus, midfoot pronation with femoral anteversion, and exhibited delayed loss of deciduous teeth, transmissive hypoacusia (managed with bilateral auricular prostheses), and psoriasis. Due to persistent lower limb alterations, she underwent hemiepiphysiodesis at 9 years of age. At the latest follow-up, she was receiving conventional treatment with calcitriol and phosphates, which was well tolerated. Her cardiac condition remained stable over time, without progression. Upon recent assessment, her FGF23 value was 110 pg/mL (normal range 20–90).

In this case, conventional treatment, while not correcting leg bowing, allowed for regular growth and reduced musculoskeletal pain without exacerbating calcification or impairing cardiac function. The management of ARHR2 with conventional treatment is challenging as calcitriol and phosphates are unable to correct abnormal phosphate concentrations, and nephrocalcinosis and secondary hyperparathyroidism may develop over time [44, 45]. However, in the context of ENPP1 deficiency, conventional treatment is generally not associated with an increased risk of arterial calcifications [45].

This case also highlights that the detection of a heart murmur in patients with hypophosphatemic rickets should raise suspicion for cardiological involvement with possible calcifications, which in turn is suggestive of ENPP1 deficiency. The systolic murmur observed in this case underscores the potential cardiovascular complications in patients with ENPP1 deficiency who survive beyond infancy. It also emphasizes that long-term auditory monitoring is essential in the follow-up of patients with ENPP1 deficiency, as hearing loss may develop progressively in these patients. Dermatologic conditions, such as punctuated keratoderma, have also been reported in patients with ENPP1 deficiency [46, 47], and psoriasis described in this case might be another associated manifestation. In summary, a combination of heart murmur, valvular calcifications, hearing impairment, various dermatological conditions, and delayed loss of deciduous teeth in a patient with hypophosphatemic rickets should prompt suspicion of ENPP1 deficiency, requiring confirmation through genetic analysis.

#### Case 5

##### Case 5

is a female patient who was born at 36 weeks of gestation in November 2023. Prenatal ultrasound showed impaired cardiac flow and hepatic hyperechogenicity, as well as cardiac effusion. Postnatal echocardiogram showed calcification and stenosis of the coronary arteries and aorta. She had pericardial effusion and severe arterial hypertension, which led to a myocardial infarction. She also presented with hypophosphatemia at birth, with phosphate values that, until the first year of life, stabilized at 1.2 mmol/L (normal range in the local laboratory for the neonatal period 1.45–2.16). She also had low PTH (4.2 pg/mL; normal range 6.5–36.8) and slightly elevated 1,25-dihydroxyvitamin D (193 pg/mL; normal range 47.8–190), with normal calcium (2.5 mmol/L) and tubular reabsorption of phosphate (97.3%). Genetic testing revealed a homozygous *ENPP1* genetic mutation c.574delG p.(Glu192Lysfs*47).

The patient was diagnosed with GACI1 and had started treatment with sodium thiosulphate, then subsequently switched to bisphosphonate therapy (i.e., etidronate). At 2 months of age, she began treatment with an investigational drug, INZ-701 (as compassionate use); these results will be presented in future publications.

##### Case 5

emphasizes the importance of early diagnosis of GACI1 to minimize adverse outcomes. In this case, the neonatologist consulted several specialists in the evaluation of the patient after the patient developed hypertension. Indeed, a multidisciplinary team comprising a sonographer, gynecologist, cardiologist, nephrologist, pediatrician, and neonatologist is essential for an accurate diagnosis. Due to the rarity of this disease, recognition and awareness of the observed signs and symptoms is important for an early and accurate diagnosis.

### Patient management pathway

#### Identification and referral

Identification and referral of patients with GACI1 or ARHR2 to a team of specialists should occur promptly upon healthcare providers suspecting a patient with ENPP1 deficiency. The types of healthcare providers involved in the identification and referral of patients with GACI1 or ARHR2 are summarized in Table 3.

**Table 3:** Healthcare providers responsible for identification and referral of patients with GACI1 or ARHR2

Patient pathway	GACI1	ARHR2
1. Disease may be suspected by:		
Gynecologist	✔	
Midwife	✔	
Neonatologist	✔	
HCP administering neonatal intensive therapy	✔	
General practitioner (primary care physician)^a^	✔	✔
Family pediatrician^a^	✔	✔
2. Patient is referred to:		
Endocrinologist		✔
Orthopedic physician		✔
Nephrologist	✔	✔
Cardiologist	✔	✔
Pediatric endocrinologist	✔	✔
3. Referrals for involvement of specialist care:		
Specialists treating rare diseases^b^	✔	✔
Geneticists	✔	

^a^In the post-neonatal period, when a suspected sign emerges that is not necessarily linked to a specific form of the disease

^b^The IRCCS Burlo Garofolo in Trieste, Italy, has a metabolic and rare diseases unit that specializes in managing patients with these diseases

Abbreviations: ARHR2: autosomal recessive hypophosphatemic rickets type 2; GACI1: generalized arterial calcification of infancy type 1; HCP: healthcare provider

For GACI1, gynecologists and midwives need to be aware of this disease because prenatal signs are present in approximately 50% of cases [30, 48]. The patient management pathway should start with prenatal and/or neonatal diagnosis for effective disease management. In newborns, the neonatologist has an essential role in the identification of the postnatal signs of GACI1 and should consult with specialists of rare diseases to determine which tests need to be conducted. A geneticist should also be consulted during the prescribing of genetic analysis and when confirming the diagnosis. For those who are treated in the neonatal intensive care unit (NICU), as well as pediatric and adult patients, cardiologists, nephrologists, and endocrinologists should also be involved in patient management.

Patients with ARHR2 should be referred by the general practitioner (i.e., primary care physician) or family pediatrician to a team of specialists (endocrinologists, orthopedic physicians, nephrologists, cardiologists, and specialized pediatricians) with experience in rare diseases (Table 3). Of note, only a few hospitals in Italy have specific units specialized in managing patients with rare diseases.

Patients with confirmed or suspected GACI1 and ARHR2 should be referred to a specialist pediatrician by the family pediatrician, who is responsible for patient care after the neonatal period. Patients should also be referred to a specialized hospital if they present with symptoms or signs that are not part of their normal growth and development or not necessarily linked to a specific disease. Formation of a multidisciplinary team involved in prenatal and postnatal care is essential for optimal management of patients with GACI1 or ARHR2.

#### Diagnostic and follow-up tests

Suspicion and clinical signs of GACI1 or ARHR2 requires exploration of the cardiovascular, skeletal, and musculo-tendinous systems with appropriate tests (Table 4). Cardiovascular tests, including X-rays, are aimed at detecting cardiovascular calcifications. Doppler ultrasound of blood vessels may also be used to screen for cardiovascular calcification, as well as arterial stenosis. Echocardiograms can detect static and dynamic alterations due to hypertensive stress, coronary disease, and valve disorders leading to heart failure. X-rays can show both vascular and extravascular calcifications visually, whereas ultrasound may identify, but not quantify, cardiac and vascular calcifications. In patients with deformities or suspected bone involvement, skeletal tests include X-rays and orthopedic examinations, as well as advanced imaging with computed tomography (CT) or magnetic resonance imaging (MRI), when clinically indicated. Patients with musculotendinous disorders are investigated for periarticular calcification and enthesopathy with X-ray and sonography. The main biomarkers to investigate the cause of these disorders and monitor disease progression are serum calcium, phosphate, calcium-phosphate product, PTH, vitamin D metabolites, and FGF23 to detect inappropriate phosphate excretion leading to hypophosphatemia and inappropriately high circulating levels of FGF23. In routine clinical practice, intact molecules of FGF23 are usually measured [49]. To accurately detect renal phosphate wasting, urine phosphate levels should be measured using the gold standard method of collecting a fasted morning urine sample and analyzing within 1 h of collection. These tests are performed in all age groups at diagnosis and may be repeated during follow-up.

**Table 4:** Medical tests for patients with GACI1 or ARHR2

Type of test		Details
Diagnostic tests	Blood biochemical tests	Calcium, phosphate, creatinine, ALP, PTH, 25(OH) vit D, FGF23
	Urinary tests	Calcium, phosphate, creatinine (to estimate GFR)
	Cardiovascular tests	ECG, BP, heart rate
	X-ray	Thorax, abdomen, upper and lower limbs
	Ultrasound	Prenatal, cardiac, and blood vessels (Doppler), abdomen, renal
	Advanced imaging	CT, MRI, CT angiography
	Secondary laboratory tests	Serum calcium, phosphate, PTH, 1,25(OH)_2_ vit D, BTM, plasma bicarbonate, FGF23, TRP, 24-hour urinary glucose, urinary LMW protein
Follow-up tests	Blood biochemical tests	Calcium, phosphate, creatinine, ALP, PTH, 25(OH) vit D, 1,25(OH)_2_ vit D, FGF23
	Urinary tests	Calcium, phosphate, creatinine
	X-ray	Thorax, abdomen, upper and lower limbs
	Ultrasound	Cardiac and blood vessels (Doppler), renal
	Other imaging	BMD, CT angiography
	Cardiovascular tests	BP, ECG
	Other	Auditory evaluation, dental examination, dermatologic examination, WOMAC, BPI, growth assessment (by measuring height/weight)

Abbreviations: 1,25(OH)_2_ vit D: 1,25-dihydroxyvitamin D; 25(OH) vit D: 25-hydroxyvitamin D; ALP: alkaline phosphatase; ARHR2: autosomal recessive hypophosphatemic rickets type 2; BMD: bone mineral density; BP: blood pressure; BPI: Brief Pain Inventory; BTM: bone turnover marker; CT: computed tomography; ECG: electrocardiogram; FGF23: fibroblast growth factor 23; GACI1: generalized arterial calcification of infancy type 1; GFR: glomerular filtration rate; LMW: low molecular weight; MRI: magnetic resonance imaging; PTH: parathyroid hormone; TRP: tubular reabsorption of phosphate; WOMAC: Western Ontario and McMaster Universities Arthritis Index

Follow-up testing depends primarily on the disease stage of GACI1 or ARHR2 and underlying diagnosis. A patient with rickets should undergo biochemical tests, urinalysis, and renal ultrasound at least once a year to monitor for treatment-related nephrocalcinosis and kidney morphology, whereas monitoring of the large vessels may require Doppler ultrasound twice a year. Patients with hypertension or cardiomyopathy should undergo cardiologic follow-up and CT angiography if complications develop or when clinically indicated. Bone mineral density could be measured every 2–3 years in patients with ARHR2. Other follow-up tests include auditory evaluation (to screen for hearing impairment), dental examination, dermatologic examination, and patient-reported outcomes, such as the Western Ontario and McMaster Universities Arthritis Index (WOMAC) and the Brief Pain Inventory (BPI). WOMAC and BPI should be conducted as a part of the general follow-up once or twice a year.

### Differential diagnosis

The diagnosis of GACI1 or ARHR2 should be confirmed by genetic tests in patients with hypophosphatemic rickets and/or vascular calcification. If these disorders are suspected, next-generation sequencing (NGS) or Sanger testing is mandatory to analyze *ENPP1* and *ABCC6* genes. In patients with hypophosphatemic rickets, the differential diagnosis should include other forms of hypophosphatemic rickets due to dentin matrix acidic phosphoprotein 1 (*DMP1*), phosphate-regulating endopeptidase homolog X (*PHEX*), and *FGF23* gene mutations. In addition, mutations at family with sequence similarity 20, member C (*Fam20C*) may cause an osteosclerotic phenotype that includes hypophosphatemia and rickets (ARHR3, Raine syndrome associated rickets) [45, 50]. Therefore, NGS sequencing for the diagnosis of ENPP1 deficiency should include *ENPP1*, *DMP1*, *PHEX*, *FGF23*, *ABCC6*, and *Fam20C*.

Misdiagnosis in adult patients may require repetition of tests. Differential diagnosis in adults can be challenging due to missing information from the perinatal period. It is important to assess the presence of tissue calcifications (e.g., in the tendons, ligaments, blood vessels, and joints) and consider the time of disease onset, which is commonly at 1 or 2 years of age in patients with hypophosphatemic rickets (may be later in some patients), but earlier in patients with GACI1. Multiple joints can be affected, with no single joint showing a clear preference [16]. In our opinion, patients should undergo joint screening for tissue calcification when symptomatic. Importantly, FGF23 levels cannot be used to differentiate between GACI1 and ARHR2 because both conditions are associated with elevated FGF23 and present with similar symptoms [22].

### Treatment

The lack of specific and effective treatments as a result of ENPP1 deficiency is a barrier to management of the disease [1]. It has been suggested that therapies targeting PPi may be beneficial to counteract pathologic calcification of soft tissue in both genetic and acquired diseases [1]. An FGF23 inhibitor, burosumab, has been useful in GACI1 and ARHR2 because FGF23 suppression leads to an increase in phosphate levels [7, 51–53]. However, due to its mechanism of action, concerns regarding the safety profile of burosumab persist, resulting in an ongoing debate in the literature.

#### GACI1

Bisphosphonate treatment has been used off-label in infants with GACI1 with the aim of inhibiting calcification; however, varying degrees of efficacy were observed in the performed studies, thus rendering their findings inconclusive [22, 33, 54]. These agents had no evident survival benefits among infants in the first 6 months of life [10], whereas a benefit in 1-year survival could occur in patients starting bisphosphonate within the first 7 days of life [10]. There is no evidence for the optimal dose or duration of bisphosphonate treatment, but their skeletal toxicity suggests avoiding the prolonged use of first-generation bisphosphonates, such as etidronate, and to discontinue their use after the resolution of calcifications [55].

The benefits of sodium thiosulfate therapy for patients with GACI1 is unclear. Sodium thiosulfate provided transient improvements in ectopic calcifications in an 11-year-old child with several deleterious *ENPP1*, *ABCC6*, and beta-globin (*HBB*) gene mutations [56]. However, in an earlier case report of a premature neonate with GACI1, there was no improvement of calcification with etidronate plus sodium thiosulfate combination therapy; the neonate passed away at 8 weeks of age [57].

Patients with cardiovascular complications, such as congestive heart failure or arterial hypertension, should receive standard cardiovascular management, including antihypertensive therapy with angiotensin-converting enzyme inhibitors or angiotensin II receptor blockers [14]. Surgical treatment has also been used to successfully manage infants with severe GACI1 and cardiovascular complications, including heart transplantation for end-stage heart failure [58] or surgical repair of severe aortic arch obstruction. Mechanical ventilation may be needed in patients with severe GACI1 and respiratory distress [29].

#### ARHR2

Conventional treatment of infants with ARHR2 typically consists of supplementation with oral phosphate and an active analog of vitamin D (calcitriol or alfacalcidol) [24, 59, 60]. Oral phosphate supplementation is associated with gastrointestinal adverse effects (e.g., abdominal pain and diarrhea) that may be mitigated by dose titration [60]. It also stimulates parathyroid gland function leading to secondary hyperparathyroidism that may evolve to tertiary hyperparathyroidism [60]. Therefore, prescription of phosphates should be well balanced to avoid excessive dosage [59]. The adverse effects of calcitriol and phosphate therapy include hypercalciuria and calcium-salt precipitation risk in the kidney as nephrocalcinosis and kidney stones, and the deterioration of kidney function to uremia [60]. The active vitamin D dose should be adjusted to avoid hypercalciuria and to provide maximal therapeutic efficacy, monitored by measuring serum ALP and phosphate, growth velocity, and leg bowing as clinical indicators [59]. This conventional treatment can reduce symptoms and correct skeletal and growth alterations, but fails to correct the primary defect of tubular phosphate reabsorption and soft tissue calcification [60]. Furthermore, several clinical studies are currently investigating the efficacy and safety of novel treatments for ENPP1 deficiency. These include NCT05734196 (a phase 1B trial evaluating the safety and tolerability of INZ-701 in infants with ENPP1 deficiency; the ENERGY study), NCT06046820 (a phase 3 study evaluating the efficacy and safety of INZ-701 in children with ENPP1 deficiency; ENERGY 3), and NCT04686175 (a recently-completed phase 1/2 trial evaluating the efficacy and safety of INZ-701 in adults with ENPP1 deficiency).

### Strengths and limitations

A key strength of this article is that, given the paucity of data on ENPP1 deficiency, it adds to the clinical evidence for managing patients with GACI1 and ARHR2. However, the study is limited by the specific clinical experience of the authors who treated only five patients. In addition, the limited numbers in the expert panel may reduce the generalizability of these findings.

## Conclusions

ENPP1 deficiency is a rare genetic condition that is associated with a broad spectrum of clinical phenotypes including, but not limited to, GACI1 and ARHR2, that have a significant burden of morbidity and mortality. Early identification of the clinical signs of disease, in combination with comprehensive diagnostic and follow-up tests, is essential for effective patient management. A diagnosis of ENPP1 deficiency should be confirmed by NGS analysis. Treatment options for ENPP1 deficiency remain limited; however, ongoing clinical trials are investigating new treatment options such as INZ-701 for patients with these rare genetic conditions. 

## Data Availability

All data generated or analyzed during this study are included in this published article.
